# Predictors of neutrophil extracellular traps markers in type 2 diabetes mellitus: associations with a prothrombotic state and hypofibrinolysis

**DOI:** 10.1186/s12933-019-0850-0

**Published:** 2019-04-16

**Authors:** Agata H. Bryk, Shannon M. Prior, Krzysztof Plens, Malgorzata Konieczynska, Jerzy Hohendorff, Maciej T. Malecki, Saulius Butenas, Anetta Undas

**Affiliations:** 1 Institute of Cardiology, Jagiellonian University Medical College, 80 Pradnicka St., 31-202 Krakow, Poland; 2 John Paul II Hospital, Krakow, Poland; 3 Department of Biochemistry, University of Vermont, Colchester, VT USA; 4 KCRI, Krakow, Poland; 5 Department of Metabolic Diseases, Jagiellonian University Medical College, Krakow, Poland; 6 Department of Metabolic Diseases, University Hospital, Krakow, Poland; 7 Faculty of Medicine and Health Sciences, Jan Kochanowski University, Kielce, Poland

**Keywords:** Type 2 diabetes, Fibrinolysis, Neutrophil extracellular traps, Fibrin clot, Cardiovascular disease

## Abstract

**Background:**

Type 2 diabetes mellitus (T2DM) is associated with a hypercoagulable state and increased neutrophil extracellular traps formation (NETosis). We investigated predictors of NETosis and cell death markers in circulating blood and their association with a prothrombotic state in T2DM.

**Methods:**

In a cross-sectional study involving 113 T2DM patients aged 63.7 ± 8.2 years, we investigated citrullinated histone H3 (H3Cit), cell-free deoxyribonucleic acid (cfDNA), myeloperoxidase, neutrophil elastase, and inflammation markers, along with thrombin generation (TG), plasma clot lysis time (CLT), clot permeability (K_s_) and fibrinolysis inhibitors.

**Results:**

On multivariate logistic regression analysis adjusted for age and gender, predictors of high H3Cit (≥ 7.36 ng/mL, upper quartile) were: glycated hemoglobin (HbA1c) ≥ 7.0% and interleukin-6. Interleukin-6 was also found to be a predictor of high cfDNA (≥ 2.84 µg/mL, upper quartile) along with glucose. Citrullinated histone H3 and cfDNA correlated positively with CLT and inversely with K_s_, while TG associated solely with cfDNA. These associations were not seen with myeloperoxidase and neutrophil elastase. Patients with previous myocardial infarction (n = 21, 18.6%) had higher H3Cit (+108%, p < 0.001) and cfDNA (+45%, p = 0.022). On multivariable analysis adjusted for potential confounders, H3Cit and cfDNA, along with plasminogen activator inhibitor-1 and concomitant cardiovascular disease, were predictors of CLT. Citrullinated histone H3 alone was a predictor of K_s_ and only cfDNA was a predictor of peak thrombin generated.

**Conclusions:**

In T2DM, NETosis detectable in circulating blood is associated with inflammatory state and a prothrombotic state, especially hypofibrinolysis.

## Background

A prothrombotic fibrin clot phenotype involves faster formation of dense meshwork composed of thinner and highly branched fibres which are relatively resistant to plasmin-induced lysis [1]. A number of diseases have been demonstrated to be associated with such unfavourable clot properties, including type 2 diabetes mellitus (T2DM) [2]. Decreased clot permeability [3] and impaired efficiency of fibrinolysis [4] in T2DM have been attributed to multiple alterations, including elevated plasminogen activator inhibitor 1 (PAI-1) [5] and thrombin activatable fibrinolysis inhibitor (TAFI) [6], increased glycation of fibrinogen [7] and plasminogen [8], and enhanced thrombin generation (TG) [9] in association with low-grade systemic inflammation [4].

There is growing evidence showing that inflammation and increased blood coagulation are in part related to each other via formation of neutrophil extracellular traps, NETosis [10]. NETs are structures composed of nuclear components such as histones and deoxyribonucleic acid (DNA), and granule constituents, such as myeloperoxidase and neutrophil elastase (NE), which disarm and kill bacteria extracellularly [11]. Addition of histones to the plasma of healthy volunteers has been demonstrated to stimulate TG [12]. In patients with coronary artery disease (CAD), double-stranded DNA, nucleosomes, citrullinated histone H4, and myeloperoxidase–DNA complexes, have been shown to positively associate with formation of thrombin-antithrombin complexes [13]. In a purified system, the addition of histone-DNA complexes to fibrinogen has been shown to result in thicker, more stable and rigid fibrin fibres and prolonged clot lysis [14]. It has been suggested that this effect may be DNA concentration-dependent [15], although in vitro NETs are considered to display largely antifibrinolytic properties [14].

Little is known about NETosis in T2DM. It has been demonstrated that in vitro, a high glucose concentration may either impair [16] or increase [17] NETs formation. Moreover, enhanced inflammation, as evidenced by elevated interleukin-6 (IL-6), promoted NETosis in vitro [16]. Increased plasma nucleosomes, extracellular DNA, and NE levels in association with glycated haemoglobin (HbA1c) have been reported in patients with T2DM compared with non-diabetic individuals [17]. To the best of our knowledge, it is unclear whether NETosis, detectable in circulating blood, could contribute to a thrombotic tendency, including the prothrombotic clot phenotype, observed in T2DM patients.

Cardiovascular disease (CVD) and diabetic kidney disease have been identified as factors associated with increased extracellular DNA in a small group of 38 patients with T2DM [17], while metformin has been found to reduce histones, DNA, and NE detectable in the blood of patients with pre-diabetes [18] or newly-diagnosed T2DM [19]. The impact of anti-diabetic agents in patients with long-lasting T2DM on NETosis markers has not been studied.

We aimed to investigate factors which determine NETosis in T2DM and the contribution of circulating markers of NETosis and cell death to a prothrombotic state in patients with T2DM.

## Methods

### Study design

In this cross-sectional study, we enrolled Caucasian patients aged ≥ 18 years who fulfilled the World Health Organization criteria for T2DM [20]. Patients were recruited in Cracow, Poland, between October 2016 and July 2017. The exclusion criteria were as follows: signs of acute infection, arterial or venous thromboembolic events within the previous 6 months, current anticoagulant therapy, known cancer, recent trauma or surgery, autoimmune diseases, and pregnancy. All subjects provided written, informed consent. The Bioethics Committee at the Jagiellonian University Medical College approved the study.

Demographic and clinical data, including the time since T2DM diagnosis, were collected at enrolment. Arterial hypertension was diagnosed if a patient met one of the following criteria: (1) a history of hypertension; (2) antihypertensive treatment prior to admission; (3) consistent systolic or diastolic pressure ≥ 140 mmHg or ≥ 90 mmHg, respectively. CVD was defined as documented stable CAD, peripheral artery disease (PAD), stroke or transient ischemic attack due to the vascular disease confirmed by imaging [21]. CAD was diagnosed in all patients who had previous myocardial infarction (MI), underwent percutaneous coronary intervention, or in all patients with signs and symptoms and positive results of either non-invasive testing or invasive coronary angiography [22]. Previous MI, ischaemic stroke, or previous revascularisation was established based on medical records. PAD was diagnosed based on symptoms with an ankle-brachial index < 0.9, and prior revascularisation [23]. For the detection of albuminuria, the threshold of albumin-to-creatinine ratio (30 mg/g) was used [24] in subjects without urinary tract infection symptoms.

### Laboratory investigations

Fasting blood samples were obtained from antecubital vein between 6 and 8 A.M. White blood cells, haemoglobin, platelet count, fasting glucose, creatinine, alanine aminotransferase, activated partial thromboplastin time (aPTT), prothrombin time and lipid profile were assayed by routine laboratory techniques. Glomerular filtration rate (GFR) was calculated using the Chronic Kidney Disease Epidemiology Collaboration equation. High-sensitivity C-reactive protein (hs-CRP) was measured by latex-enhanced turbidimetric immunoassay using a Cobas 6000 analyser (Roche Diagnostics GmbH, Mannheim, Germany; Hitachi High-Technologies Corporation, Tokyo, Japan). HbA1c was measured by high-performance liquid chromatography using the Variant II Turbo analyser (Hercules, CA, USA).

### NETs components

Commercially-available ELISA kits were used to quantify citrullinated histone H3 (H3Cit, Cayman Chemical, Ann Arbor, MI, USA), neutrophil elastase (NE, Abcam, Cambridge, MA, USA), myeloperoxidase, IL-6, and interleukin 8 (IL-8, R&D Systems, Minneapolis, MN, USA). Concentration of cfDNA was measured using the assay kit (Invitrogen, Life Technologies, CA, USA) according to manufacturer’s instructions. High H3Cit and high cfDNA were defined as H3Cit and cfDNA, respectively, in the upper quartile.

### Coagulation and fibrinolysis parameters

To obtain citrated plasma, blood samples were mixed with 3.2% sodium citrate (9:1), centrifuged for 20 min and stored at − 80 °C. Fibrinogen was determined with the von Clauss method. Plasminogen and α2-antiplasmin were measured by chromogenic assays (Siemens, Munich, Germany). Commercially available immunoenzymatic assays were used to measure plasma PAI-1 antigen (Hyphen, Neuville-sur-Oise, France), TAFI (Hyphen, Neuville-sur-Oise, France), thrombomodulin (Diagnostica Stago, Parsippany, NJ, USA), P-selectin and platelet factor 4 (R&D Systems, Minneapolis, MN, USA).

### Calibrated automated thrombogram (CAT)

Assessment of the TG profile was performed as previously described [25]. Citrated plasma samples were thawed at 37 °C for 3 min and 5 mg/mL corn trypsin inhibitor was immediately added to achieve a 0.1 mg/mL final concentration. Eighty μL of each plasma sample was added to a 96-well plate followed by addition of relipidated tissue factor [26] at a final concentration of 5 pM. The fluorogenic substrate used was benzyloxycarbonyl-Gly-Gly-Arg-7-amido-4methyl-coumarin·HCl (Z-GGR-AMC) (Bachem, Torrance, CA, USA). Twenty millilitre of a 2.5 mM Z-GGR-AMC/90 mM CaCl_2_ solution in HEPES-buffered saline was added to plasma samples to achieve final concentrations of 417 μM and 15 mM, respectively. A 3 min incubation period at 37 °C followed to allow recalcification of the plasma. Twenty millilitre of a 120 μM phospholipid vesicle solution (25% dioleoyl-*sn*-glycero-3-phospho-l serine and 75% 1,2-dioleoyl-*sn*-glycero-3-phosphocholine) (Avanti Polar Lipids, Inc, Alabaster, Al) in HEPES-buffered saline was then added to plasma samples to achieve a final concentration of 20 μM, thus initiating TG. Fluorescence readings began immediately and hydrolysis of the AMC (7-amino-4-methylcoumarin) substrate (at 370 nm excitation and 460 nm emission wavelengths) was followed over a 3600 s period. Changes in fluorescence were converted to thrombin concentration using a calibration curve built by sequential dilutions of human thrombin (Haematologic Technologies, Inc., Essex Junction, VT). Human thrombin was produced in-house [27]. The plate reader used was the BioTek Synergy 4 and analysis was performed using the Gen5 plate reader software (BioTek, Winooski, VT, USA).

### Clot permeability and clot lysis time (CLT)

Fibrin clot permeability was determined as previously described [28, 29]. Briefly, 20 mmol/L calcium chloride and 1 U/mL human thrombin were added to 120 µL of citrated plasma. After incubation in a wet chamber for 120 min, tubes containing the clots were connected to a reservoir of a Tris buffer pH 7.5, and after washing, flow rates of buffer through the fibrin clots were measured by timing the permeation of consecutive drops through each tube within 60 min and recording the weight of each drop for exact volume. A permeation coefficient (K_s_), which indicates the size of fibrin clot pores, was calculated from the following equation: K_s_ = Q×L × η/t × A×Δp, where Q is the flow rate in time (t); L, the length of a fibrin gel; η, the viscosity of liquid (in poise); A, the cross-sectional area (in square centimetres), and Δp, a differential pressure (in dynes per square centimetre).

CLT was measured as described [30]. Briefly, citrated plasma was mixed with 15 mM calcium chloride, 10,000×-diluted human tissue factor (Innovin, Siemens), with a final concentration of 0.6 pM, 12 µmol/L phospholipid vesicles (highly purified phosphatidylcholine, phosphatidylserine, and sphingomyelin from Rossix, Mölndal, Sweden), and 60 ng/mL recombinant tissue plasminogen activator (Boehringer Ingelheim, Ingelheim, Germany). The mixture was transferred to a microtiter plate and its turbidity was measured at 405 nm at 37 °C. Clot lysis time was defined as the time from the midpoint of the clear-to-maximum-turbid transition, which represents clot formation, to the midpoint of the maximum-turbid to-clear transition (representing the lysis of the clot).

### Statistical analysis

Categorical variables were presented as numbers (percentages) and compared by Fisher’s exact test for 2 × 2 contingency tables (if 20% of cells have expected count less than 5), Pearson’s chi-squared test was used otherwise. Continuous variables were expressed as mean ± standard deviation or median (interquartile range). Normality was assessed by the Shapiro–Wilk test. Equality of variances will be assessed using the Levene’s test. NETs markers were dichotomized into 2 groups by using an upper quartile split. Differences between groups were compared using the Student’s or the Welch’s *t* test depending on the equality of variances for normally distributed variables. The Mann–Whitney U test was used for comparison of two non–normally distributed continuous variables, while more groups were compared using the Kruskal–Wallis test. Post-hoc comparisons were made using the Steel–Dwass method. The association between two continuous variables was assessed by Pearson’s or Spearman’s rank correlation. The odds ratio of high H3Cit and cfDNA were determined by multivariate forward regression and presented with 95% confidence interval (95% CI). To study determinants of TG, CLT and K_s_, univariate and multivariate regression analyses were performed. Multivariate models were fitted using backward stepwise regression with the p < 0.05 threshold stopping rule. If variables correlated with r ≥ 0.5, only one of them was included in the multivariate model. Receiver operating characteristic curves and the area under the curve (AUC) were used to analyse the discriminatory power of CLT with respect to CVD. Two-sided p-values < 0.05 were considered statistically significant.

The study was powered to have a 80% chance of detecting a 30% difference in cfDNA using a significance level of 0.05, based on the values of cfDNA in T2DM patients in the previous study [17]. To demonstrate such a difference or greater, 20 patients or more were required in each group.

All calculations were performed with JMP^®^, Version 14.0.0 SAS Institute Inc., Cary, NC.

## Results

The final analysis included 113 T2DM patients, 59 (52.2%) men and 54 (47.8%) women, aged between 39 and 79 years (mean 63.7 ± 8.2 years). Sixty (53.1%) patients were treated with oral hypoglycaemic drugs, 32 (28.3%) with insulin and oral drug, 13 (11.5%) with insulin, and 8 (7.1%) patients had only dietary therapy. HbA1c levels ranged from 5.1 to 12.1% (median 6.9%, 52 mmol/mol). Median time since T2DM diagnosis was 7.0 (3.0-15.0) years. Among 53 (46.9%) patients with CVD, there were 21 (18.6%) with previous MI, 10 (8.9%) with PAD, and 5 patients (4.4%) suffered from stroke or transient ischemic attack in the past. As expected, H3Cit correlated with cfDNA (r = 0.53, p < 0.001). The two markers positively associated with myeloperoxidase (r = 0.36, p < 0.001 and r = 0.26, p = 0.006) but not with NE.

### Associations with patient characteristics

**Fig. 1:**
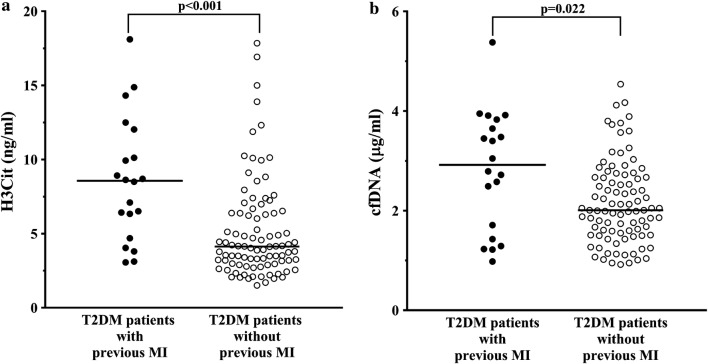
Citrullinated histone 3 (H3Cit, **a**) and cell-free deoxyribonucleic acid (cfDNA, **b**) in type 2 diabetes mellitus (T2DM) patients with previous myocardial infarction (MI, black dots) and the remainder (white dots). p-values were computed using the Mann–Whitney U test. Data on H3Cit and cfDNA levels were missing for one patient. Horizonal lines represent the median in each group

Gender, BMI, and smoking did not associate with NETosis markers. Patients with high H3Cit, defined as ≥ 7.36 ng/mL (upper quartile), did not differ from the remainder with regard to demographic data and comorbidities, except for MI being more prevalent among patients with high H3Cit (Table 1). This was also the case for patients with high cfDNA, defined as ≥ 2.84 µg/mL (upper quartile). Patients following MI had higher H3Cit (+108%; 8.57 [5.52–11.08] vs. 4.13 [2.97–6.39] ng/mL, p < 0.001, Fig. 1a) and higher cfDNA (+45%; 2.92 [1.57–3.74] vs. 2.01 [1.53–2.67] µg/mL, p = 0.022, Fig. 1b) when compared with the remainder. Median time from the MI was 7.0 (2.2–12.0) years. There was an inverse correlation between cfDNA and time since MI (r = − 0.69, p = 0.001). Regarding microangiopathic complications, patients with and without albuminuria did not differ in terms of circulating markers of NETosis (data not shown).

**Table 1:** Comparison of patient characteristics in relation to citrullinated histone 3 (H3Cit) and cell-free deoxyribonucleic acid (cfDNA)

Variable	Patients with H3Cit ≥ 7.36 ng/mL (n = 28)	Patients without H3Cit < 7.36 ng/mL (n = 84*)	p-value	Patients with cfDNA ≥ 2.84 µg/mL (n = 28)	Patients with cfDNA < 2.84 µg/mL (n = 84*)	p-value
Demographic data						
Age, years	64.6 ± 7.1	63.5 ± 8.6	0.52	63.1 ± 7.4	64.0 ± 8.5	0.63
Male gender, n (%)	11 (39.3)	47 (55.9)	0.12	13 (46.4)	45 (53.6)	0.51
BMI, kg/m^2^	30.5 (27.4–37.8)	32.5 (29.6–36.8)	0.11	32.1 (29.5–37.7)	32.0 (29.1–36.2)	0.61
Type 2 diabetes data						
HbA1c, %	7.55 (6.23–8.58)	6.80 (6.00–8.20)	0.18	7.80 (6.73–8.90)	6.70 (6.00–8.00)	0.006
HbA1c, mmol/mol	57.0 (43.0–69.4)	51.0 (42.0–66.1)	0.29	61.0 (50.0–70.5)	50.0 (41.0–64.0)	0.010
Time since type 2 diabetes diagnosis, years	7.5 (5.0–17.5)	7.0 (2.3–12.8)	0.16	7.0 (5.0–18.5)	7.0 (2.5–12.0)	0.19
Comorbidities, n (%)						
Current or former smoking	14 (50.0)	48 (56.5)	0.66	13 (46.4)	49 (57.6)	0.38
Arterial hypertension	27 (96.4)	77 (91.7)	0.68	27 (96.4)	77 (91.7)	0.68
Cardiovascular disease	16 (57.1)	36 (42.9)	0.19	15 (53.6)	37 (44.1)	0.38
Previous myocardial infarction	11 (39.3)	9 (10.7)	< 0.001	10 (35.7)	11 (12.9)	0.004
Family history of cardiovascular disease	9 (32.1)	21 (25.0)	0.46	4 (14.3)	26 (31.0)	0.63
Heart failure	4 (14.3)	4 (4.8)	0.11	3 (10.7)	5 (6.0)	0.41
GFR ≤ 60 mL/min/1.73 m^2^	4 (14.3)	12 (14.5)	1.0	6 (21.4)	10 (12.1)	0.22
ACR ≥ 30 mg/g creatinine	6 (21.4)	15 (17.9)	0.74	3 (10.7)	18 (21.2)	0.18
Medication, n (%)						
Aspirin	18 (64.3)	55 (65.5)	0.91	19 (67.9)	54 (64.3)	0.73
Clopidogrel	4 (14.3)	5 (5.9)	0.22	4 (14.3)	5 (5.9)	0.22
β-blocker	20 (71.4)	61 (72.6)	0.90	21 (75.0)	60 (71.4)	0.71
Statin	19 (67.9)	55 (65.5)	0.82	19 (67.9)	55 (65.5)	0.81
ACEI	16 (57.1)	46 (54.8)	0.83	18 (64.3)	44 (52.3)	0.27
Angiotensin receptor blocker	11 (39.3)	24 (28.6)	0.29	8 (28.6)	27 (32.1)	0.72
Calcium antagonist	15 (53.6)	33 (39.3)	0.19	13 (46.4)	35 (41.7)	0.66
Thiazide diuretics	10 (35.7)	26 (30.9)	0.64	9 (32.1)	27 (32.1)	1.0
Indapamide	5 (17.9)	21 (25.0)	0.44	5 (17.9)	21 (25.0)	0.44
Loop diuretics	7 (25.0)	14 (16.7)	0.33	7 (25.0)	14 (16.7)	0.33
Hypoglycemic treatment, n (%)						
No hypoglycemic drugs	0 (0)	8 (9.5)	0.20	1 (3.6)	7 (8.3)	0.68
Oral drug	16 (57.1)	44 (52.4)	0.39	15 (53.5)	45 (53.6)	0.78
Insulin	3 (10.8)	9 (10.7)	0.66	3 (10.8)	9 (10.7)	0.66
Insulin and oral drug	9 (32.1)	23 (27.4)	0.63	9 (32.1)	23 (27.4)	0.64
Laboratory investigations						
White blood cells count, × 10^6^/L	6.77 (5.72–8.87)	7.32 (6.45–8.49)	0.36	6.72 (5.78–8.17)	7.32 (6.45–8.54)	0.19
Hemoglobin, g/dL	13.4 (12.5–14.2)	13.9 (13.0–14.7)	0.03	13.4 (12.5–14.0)	14.0 (130–14.8)	0.014
Platelet count, × 10^9^/L	227.5 (198–270)	230.0 (185–272)	0.94	2167 (191–251)	232 (186–278)	0.42
Fasting glucose, mmol/L	7.40 (6.63–8.80)	7.14 (5.82–8.44)	0.28	8.07 (7.05–11.11)	6.70 (5.80–7.90)	< 0.001
GFR, mL/min/m^2^	86.5 (73.0–92.5)	82.0 (68.5–92.0)	0.42	80.5 (67.0–92.0)	84.5 (71.0–92.5)	0.17
Alanine aminotransferase, U/L	24.0 (18.0–37.5)	26.5 (21.0–40.8)	0.30	27.5 (20.0–39.0)	26.0 (20.0–40.0)	0.58
Total cholesterol, mmol/L	3.94 (3.45–5.48)	4.26 (3.50–5.27)	0.79	4.09 (3.76–5.12)	4.23 (3.40–5.50)	0.85
LDL-cholesterol, mmol/L	2.29 (1.75–3.81)	2.54 (1.17–3.43)	0.80	2.40 (1.76–3.41)	2.57 (1.80–3.57)	0.39
HDL-cholesterol, mmol/L	1.35 (1.09–1.49)	1.14 (0.98–1.45)	0.11	1.22 (1.00–1.45)	1.15 (1.03–1.48)	0.76
Triglycerides, mmol/L	1.49 (1.06–1.87)	1.54 (1.19–2.10)	0.29	1.74 (1.26–5.62)	1.48 (1.16–1.89)	0.12
C-reactive protein, mg/L	2.90 (1.09–4.75)	2.33 (1.20–5.14)	0.94	3.43 (1.26–5.62)	2.33 (1.11–4.22)	0.26

Values are given as mean ± SD or median (interquartile range)

BMI, body mass index; HbA1c, glycated hemoglobin; GFR, glomerular filtration rate; ACR, albumin-to-creatinine ratio; ACEI, angiotensin-converting-enzyme inhibitor; LDL, low-density lipoprotein; HDL, high-density lipoprotein

* Data on H3Cit and cfDNA levels were missing for one patient

Patients treated with metformin had longer time since T2DM diagnosis (8.0 [4.0–15.0] vs. 5.0 [0.4–9.5] years, p = 0.020), increased H3Cit (4.70 [3.35–8.23] vs. 3.50 [2.21–6.22] ng/mL, p = 0.010) and prolonged CLT (97.42 ± 19.26 vs. 87.59 ± 17.39 min, p = 0.020), irrespective of HbA1c and fasting glucose levels, which were comparable among those two groups of patients. Conversely, cfDNA was unaffected by metformin therapy. Treatment with aspirin or statin was not associated with lower H3Cit or cfDNA (data not shown).

**Fig. 2:**
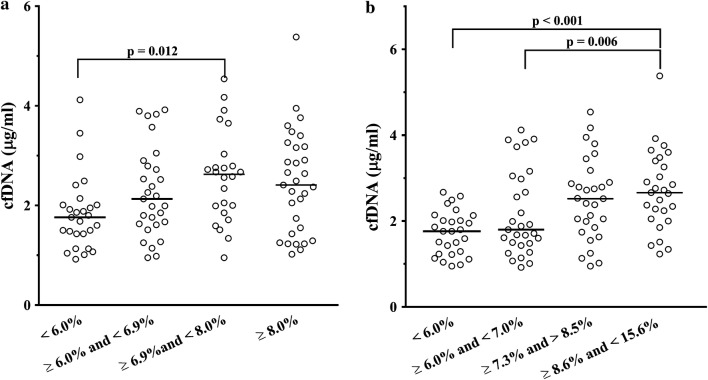
Cell free DNA (cfDNA) in patients with glycated haemoglobin (HbA1c) categorised in 4 quartiles (**a**), and in patients with fasting glucose categorised in 4 quartiles (**b**). Groups were compared using the Kruskal–Wallis test, p-values were computed using the Steel–Dwass method. Data on cfDNA levels were missing for one patient. Horizonal lines represent the median in each group

**Fig. 3:**
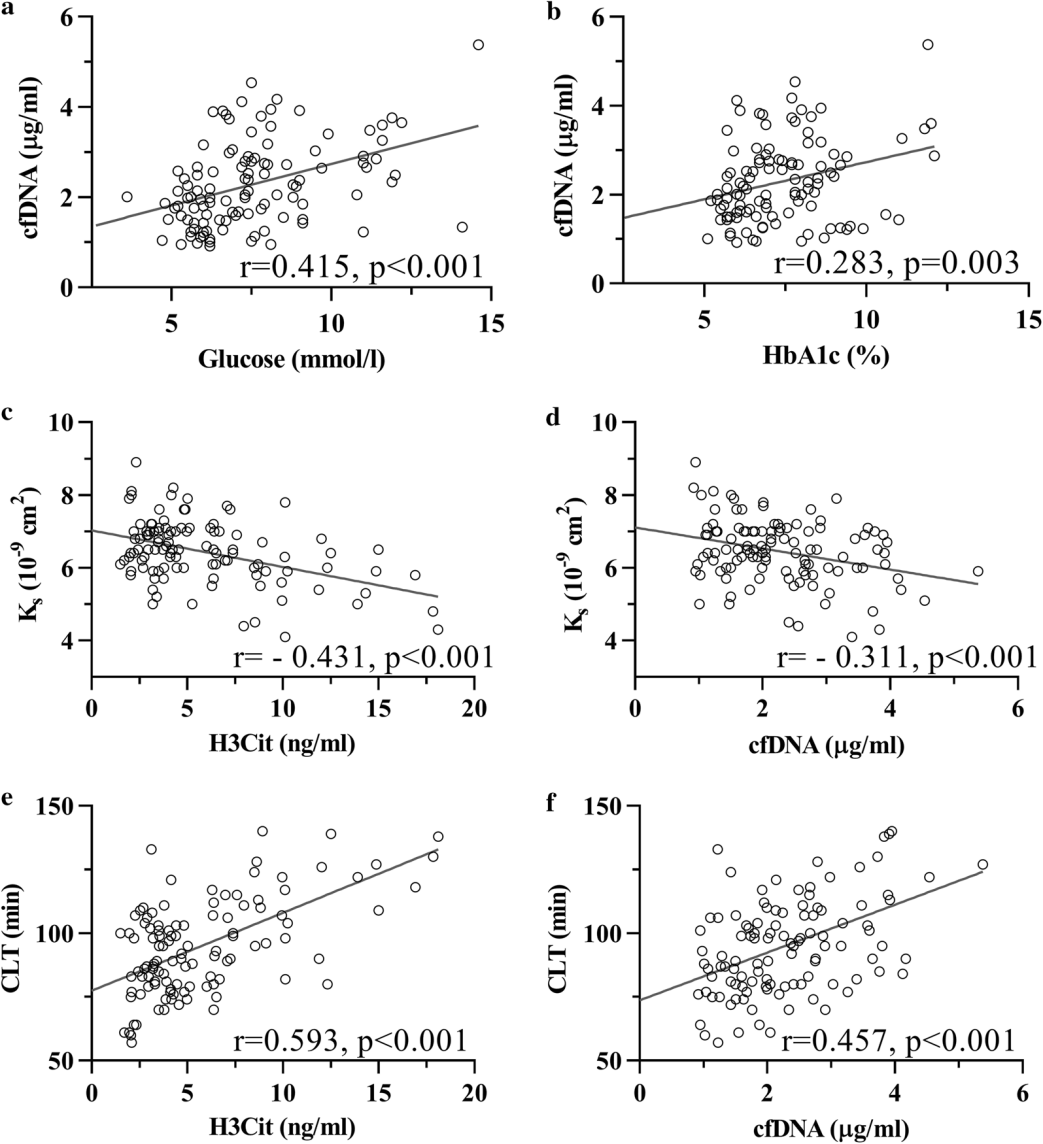
Correlations between fasting glucose and cell-free deoxyribonucleic acid (cfDNA, **a**), glycated haemoglobin (HbA1c) and cfDNA (**b**), citrullinated histone 3 (H3Cit) and clot lysis time (CLT, **c**), cfDNA and CLT (**d**), H3Cit and clot permeability (K_s_, **e**), and cfDNA and K_s_ (**f**). Correlation coefficients were computed using Spearman’s rank correlation test. Data on H3Cit and cfDNA levels were missing for one patient

Patients with high H3Cit did not differ in regard to routine laboratory test results compared with the remainder (Table 1). Higher HbA1c (+15.6%) and fasting glucose (+20.4%) were observed in patients with high cfDNA when compared with the rest of the study group (Table 1). Cell-free DNA was higher in patients with HbA1c in the third quartile when compared to those in the first quartile (+49.4%, p = 0.012, Fig. 2a). Concentrations of cfDNA were higher in patients with fasting glucose in the third and fourth quartiles compared with those in the first quartile (+43.2%, p < 0.001 and +51.1%, p = 0.006, respectively, Fig. 2b). cfDNA correlated with fasting glucose (r = 0.415, p < 0.001, Fig. 3a) and with HbA1c (r = 0.283, p = 0.003, Fig. 3b). HbA1c ≥ 8.0% was associated with approximately 2.5-fold greater odds of having high cfDNA (OR 2.55, 95% CI 1.04–6.29, p = 0.040).

### Predictors of H3Cit and cfDNA

On multivariate logistic regression analysis adjusted for age and gender, predictors of high H3Cit in T2DM patients were: HbA1c ≥ 7.0% (OR 5.21, 95% CI 1.34–24.83, p = 0.016) and IL-6 (OR 2.40, 95% CI 1.76–3.61, p < 0.001). Interleukin-6 was also found to be a predictor of high cfDNA (OR 1.60, 95% CI 1.27–2.10, p < 0.001) along with glucose (OR 1.62, 95% CI 1.27–2.14, p < 0.001).

### Associations with coagulation, fibrinolysis, and inflammatory markers

H3Cit correlated weakly with fibrinogen, plasminogen, PAI-1, and TAFI (all r < 0.25, all p < 0.05; Table 2). cfDNA showed similar weak correlations with PAI-1 and TAFI. Patients with high H3Cit trended to have slightly higher thrombomodulin (+7%, Table 2). Importantly, H3Cit and cfDNA inversely correlated with K_s_ (r = − 0.431 and r = − 0.381, both p < 0.001, Fig. 3c, d), while there were strong positive associations of both H3Cit and cfDNA with CLT (r = 0.590 and r = 0.457, both p < 0.001, Fig. 3e, f, Table 2). cfDNA, but not H3Cit, correlated with peak thrombin (r = 0.391, all p < 0.001, Table 2), but not with other TG parameters. Myeloperoxidase correlated with CLT (r = 0.253, p = 0.007), but not with K_s_ or TG. As expected, both H3Cit and cfDNA correlated with IL-6 and IL-8 (all p < 0.001), with the highest coefficient observed between H3Cit and IL-6 (r = 0.711, Table 2). Myeloperoxidase, but not NE, associated with IL-6 and IL-8 (r = 0.306 and r = 0.372, both p < 0.001).

**Table 2:** Comparison of coagulation and fibrinolysis proteins and clot phenotype parameters in patients in relation to citrullinated histone 3 (H3Cit) and cell-free deoxyribonucleic acid (cfDNA)

Variable	Patients with H3Cit ≥ 7.36 ng/mL (n = 28)	Patients without H3Cit < 7.36 ng/mL (n = 84*)	p-value	Patients with cfDNA ≥ 2.84 µg/mL (n = 28)	Patients with cfDNA < 2.84 µg/mL (n = 84*)	p-value
Coagulation and fibrinolysis components						
Fibrinogen, g/L	3.54 (3.20–4.02)	3.45 (3.09–3.85)	0.17	3.56 (3.17–3.87)	3.44 (3.10–3.9)	0.44
α2-antiplasmin, %	106.0 (102.0–112.0)	103.0 (96.0–110.0)	0.07	104.5 (96.0–111.5)	104.0 (98.0–110.0)	0.88
Plasminogen, %	110.0 (101.5–118.5)	108.0 (99.0–120.0)	0.55	109.5 (97.5–127.5)	109.0 (100.0–118.8)	0.75
PAI-1, ng/mL	36.4 (32.0–45.4)	35.0 (30.6–40.9)	0.19	37.6 (33.0–44.8)	35.0 (30.8–40.9)	0.13
TAFI, %	104.0 (96.5–119.3)	101.5 (90.0–114.0)	0.06	109.0 (93.5–120.5)	102.0 (90.0–113.8)	0.07
Endothelial injury marker						
Thrombomodulin, ng/mL	3.02 (2.45–3.80)	2.80 (2.35–3.11)	0.056	2.95 (2.47–3.73)	2.74 (2.33–3.19)	0.13
Interleukins						
Interleukin 6, pg/mL	7.19 (5.17–8.78)	3.02 (2.19–4.63)	< 0.001	6.14 (3.25–8.46)	3.52 (2.22–5.19)	< 0.001
Interleukin 8, pg/mL	8.26 (6.08–9.37)	4.28 (3.29–6.27)	< 0.001	7.72 (4.83–9.22)	4.73 (3.61–6.79)	< 0.001
Thrombin generation						
Lag phase, s	1053 (763–1343)	1004 (777–1350)	0.82	897 (762–1182)	1,050 (789–1383)	0.13
Peak thrombin, nmol	115.7 (90.0–166.6)	116.8 (87.7–153.5)	0.45	142.3 (100.0–188.0)	112.5 (86.6–147.2)	0.012
Time-to-peak, s	1369 (1067–2035)	1395 (1128–1889)	0.94	1184 (1019–1559)	1417 (1149–1892)	0.08
ETP, nM × s	104,732 (92,039–122,405)	103,810 (83,751–126,823)	0.93	111,864 (94,181–134,420)	103,810 (81,390–121,805)	0.26
Platelet markers						
P-selectin, ng/mL	62.9 (46.1–88.1)	72.4 (52.4–91.7)	0.45	70.7 (47.0–88.4)	68.1 (51.43–91.7)	0.63
Platelet factor 4, ng/mL	80.5 (64.8–89.6)	82.0 (70.7–90.8)	0.40	80.5 (72.2–88.7)	82.6 (68.9–91.3)	0.45
Clot permeability						
K_s_, × 10^−9^ cm^2^	5.90 (5.15–6.40)	6.65 (6.20–7.10)	< 0.001	6.20 (5.58–6.88)	6.50 (6.00–7.00)	0.022
Clot lysis time, min	114.0 (99.3–126.8)	87.0 (78.3–100.0)	< 0.001	103.5 (90.0–125.0)	89.0 (79.0–103.0)	0.001

Values are given as mean ± SD or median (interquartile range)

PAI-1, plasminogen activator inhibitor 1; TAFI, thrombin activatable fibrinolysis inhibitor; ETP, endogenous thrombin potential; K_s_, Darcy’s constant (permeability coefficient)

* Data on H3Cit and cfDNA levels were missing for one patient

### Predictors of thrombin generation, clot density and lysis

In univariate linear regression analysis, cfDNA explained 15.3% of the variation in peak thrombin generated in T2DM patients, while the corresponding value for H3Cit was negligible (about 1%). In multivariate regression analysis adjusted for age, gender, and fibrinogen, cfDNA was a predictor of peak thrombin generated (Table 3). In univariate regression analysis, H3Cit and cfDNA accounted for < 1% of the variation in AUC (data not shown), while for 18.6% and 9.7% of the variation in K_s_ respectively. In a multivariate model adjusted for age, gender, and fibrinogen, H3Cit, but not cfDNA, was found to be an independent predictor of K_s_ (Table 3).

**Table 3:** Univariate and multivariate models for predictors of peak thrombin, clot permeability (K_s_) and clot lysis time (CLT)

Predictors	U β (95% CI)	p-value	M β (95% CI)*	p-value
Peak thrombin				
cfDNA	40.83 (22.65 to 59.02)	< 0.001	17.10 (7.24 to 26.95)	< 0.001
Metformin	12.68 (− 34.07 to 8.71)	0.24	− 14.5 (− 25.20 to − 3.85)	0.008
BMI	1.23 (− 2.13 to 4.59)	0.47	1.86 (0.24 to 3.48)	0.025
K_s_				
H3Cit	− 0.10 (− 0.14 to − 0.06)	< 0.001	− 0.08 (− 0.11 to − 0.04)	< 0.001
CLT				
H3Cit	3.06 (2.28 to 3.85)	< 0.001	2.70 (1.93 to 3.46)	< 0.001
PAI-1	1.00 (0.52 to 1.46)	< 0.001	0.58 (0.17 to 0.99)	0.006
CVD	7.09 (3.73 to 10.46)	< 0.001	3.91 (1.05 to 6.76)	0.008
Time since T2DM diagnosis ≥ 10 years	4.26 (0.64 to 7.88)	0.022	3.15 (0.30 to 6.01)	0.031
CLT				
cfDNA	9.38 (5.93 to 12.83)	< 0.001	8.26 (5.04 to 11.48)	< 0.001
PAI-1	1.00 (0.52 to 1.46)	< 0.001	0.80 (0.37 to 1.23)	< 0.001
CVD	7.09 (3.73 to 10.46)	< 0.001	5.46 (2.49 to 8.43)	< 0.001

BMI, body-mass index; PAI-1, plasminogen-activator inhibitor type 1; T2DM, type 2 diabetes mellitus; CVD, cardiovascular disease

Data are presented as regression coefficients derived from the univariate and multivariate regression models. Due to the strong correlation between citrullinated histone 3 (H3Cit) and cell-free deoxyribonucleic acid (cfDNA), two separate multivariate models for CLT were provided

* Adjusted for age, gender and fibrinogen

In our patients, H3Cit explained 35.2% and cfDNA accounted for 20.9% of the variation in CLT, while the corresponding values for PAI-1 and TAFI were 13.8% and 3.6%, respectively. In multivariate linear regression analysis adjusted for age, gender, and fibrinogen, both H3Cit and cfDNA were predictors of CLT, alongside with PAI-1 and concomitant CVD (Table 3). Of note, time since diagnosis of T2DM ≥ 10 years contributed to CLT in a regression model incorporating H3Cit.

### Associations with CVD

When patients with T2DM and CVD were analysed separately, we observed longer CLT compared to subjects without CVD (102.50 [87.00–116.50] vs. 86.50 [77.50–99.00] minutes, p < 0.001). Prolonged CLT ≥ 100 min was associated with approximately 4.7-fold higher risk of concomitant CVD (OR 4.67, 95% CI 2.03–10.76, p = 0.002). CLT ≥ 100 min could be identified with H3Cit value of ≥ 7.4 ng/mL (OR 11.00, 95% CI 3.89–31.14, p < 0.001, AUC 0.71) and with cfDNA value of ≥ 2.53 µg/mL (OR 4.38, 95% CI 1.91–10.04, p < 0.001, AUC 0.67). T2DM patients with CVD did not differ from patients without CVD in regard to TG parameters and K_s_.

## Discussion

Our study is the first to investigate factors which determine circulating markers of NETosis and cell death in the largest group of T2DM patients to date, along with their associations with a prothrombotic state and fibrinolysis in this disease. The principal finding of this study is that elevated circulating markers of NETosis are associated with enhanced peak thrombin generation, decreased clot permeability, and impaired fibrinolysis in patients with T2DM. Our findings suggest that in T2DM patients, H3Cit and cfDNA could be regarded as two of the previously unknown contributors to the unfavourable clot phenotype, in addition to fibrinolysis inhibitors such as PAI-1 and TAFI [4]. Importantly, T2DM patients with prior MI are characterized by especially elevated markers of NETosis. We also found that glycaemic control, and inflammatory state reflected by IL-6, are related to NETosis in T2DM patients, indirectly modulating prothrombotic alterations in blood. The present study yields new insights into the determinants of NETosis in T2DM and its impact on thrombotic tendency, which may have clinical implications.

Our study provided several factors linked with increased circulating levels of cfDNA and H3Cit. We have extended previous reports on a positive correlation between HbA1c and nucleosomes or dsDNA [17] by showing that HbA1c exceeding 8.0% is associated with almost threefold higher odds of detecting high cfDNA in the blood of T2DM patients. Although DNA is a major component of NETs, it can be also released from cells other than neutrophils during the process of cell death. Therefore, cfDNA should be regarded as a less specific marker of NETosis when compared with H3Cit, result of enzyme peptidyl arginine-deiminase 4 activity in the early phase of NETs formation [31, 32]. To the best of our knowledge, our study is the first to show that circulating H3Cit in T2DM patients is increased in patients with HbA1c above 7.0%, after adjusting for IL-6, a potent inducer of NETs as evidenced by Joshi et al. [16]. In the present study, higher cfDNA concentrations were observed in patients with elevated fasting glycaemia, which is in line with documented in vitro direct stimulating effects of hyperglycaemia on NETs release [17]. Higher circulating H3Cit concentrations in patients on metformin without differences in cfDNA are surprising given the data on its normalizing effect on NET levels reported by Carestia et al. [19]. However, there were differences in the study design and patient characteristics between that study and ours. Firstly, the mean age of patients studied by Carestia et al. was 50 years, they had newly diagnosed T2DM, and none of them had thrombotic event during follow-up (thrombotic events in the past were not presented), whereas the mean age of the patients enrolled in our study was approximately 64 years, their time since T2DM was 7 years (interquartile range 3–15 years), and 21 (18.6%) patients had previous MI, indicating higher cardiovascular risk in the current study. It might be hypothesized that higher H3Cit levels in metformin-treated patients reflect long-term T2DM associated with effects of aging, cardiovascular diseases and poor diabetic control. In our study, patients treated with metformin had longer time since T2DM diagnosis compared with the subjects not receiving this agent. Neutrophil-associated prothrombotic effects may sustain in diabetic patients with longer disease duration (the so-called “prothrombotic memory”) as demonstrated in the study by Konieczynska et al. [28]. Those effects involved enhanced oxidative stress, endothelial injury, increased thrombin formation. The contribution of NETosis to the “prothrombotic memory” in long-standing T2DM should be further explored.

Unexpectedly, we observed a significant impact of NETosis on efficiency of fibrinolysis assessed using a global lysis test in T2DM patients. In our study, an elevation of H3Cit by 1 ng/mL was associated with an increase in CLT by 2.7 min, while an elevation of cfDNA by 1 µg/mL was associated with increase of CLT by 8.3 min. This might suggest that despite inconsistent effects of histones and cfDNA on fibrin clot lysis in purified systems [14, 15], in T2DM with concomitant inflammatory state, H3Cit and cfDNA might contribute to hypofibrinolysis. In an in vitro study, addition of histones to fibrin influenced clot structure, resulting in denser fibrin clot and thicker fibrin fibres [14]. T2DM is a typical disease in which the prothrombotic fibrin clot phenotype has been observed [33]. In ex vivo studies, a key measure of plasma clot structure is its permeability, reflected by the Darcy constant (K_s_) [33]. Our original observation is that H3Cit is an independent predictor of clot permeability in T2DM. In multivariate analysis, cfDNA did not contribute to K_s_, supporting in vitro observations showing that the effects of DNA on clot structure are minor [14].

Of note, previous MI was found in our study to be a key clinical factor associated with elevated H3Cit, cfDNA, and CLT among T2DM patients. Recent data has strongly supported the role of NETs in atherosclerosis and atherothrombosis [34], including mediating MI in the mouse model [35]. MI is a manifestation of coronary atherosclerosis, which has been reported to be associated with elevated plasma biomarkers of NETs such as double-stranded DNA, nucleosomes, and myeloperoxidase–DNA complexes [13]. Increased extracellular DNA has been observed in T2DM patients with CVD when compared to those without CVD [17]. Consistent with the theory of early NETS as an early biomarker of tissue injury in myocardial infarction [36], we observed an inverse correlation between cfDNA and time since MI. Boristoff et al. demonstrated that myeloperoxidase–DNA complexes predicted the occurrence of major adverse cardiac events (MACE), however, patients with T2DM accounted for a small proportion of patients with MACE [13]. Our study extends these previous observations by suggesting that biomarkers of NETosis could be potential candidates for further evaluation in the prediction of MACE within the subset of diabetic patients. Since we found that H3Cit ≥ 7.4 ng/mL was associated with 11-fold increased odds of prolonged CLT, it might be speculated that H3Cit measured in blood could be regarded as an additional marker of increased cardiovascular risk in T2DM. It cannot be excluded that H3Cit, via prolonged CLT, also contributes to an increased risk of venous thromboembolism in T2DM, although data regarding the impact of T2DM on venous thrombosis are inconsistent [37].

It has been previously shown that in patients with CAD, markers of cell death and NETs formation were positively associated with TG [13]. TG has been suggested to contribute to the hypercoagulable state in T2DM to a smaller extent than impairment of fibrinolysis [38, 39]. We found that cfDNA may predict peak thrombin measured using the CAT assay, with no impact on endogenous thrombin potential. Extracellular histones have been demonstrated to promote TG through platelet-dependent mechanisms [12]. It is possible that the use of platelet-poor-plasma in our study explains the weak effect of NETosis on thrombin formation in this assay. It remains to be established whether platelet-rich plasma obtained from T2DM patients may result in more potent associations between NETosis markers and TG potential. Although patients with high H3Cit trended to have slightly higher thrombomodulin, this concentration was likely insufficient to mediate enhancement of plasma TG as observed in purified systems [40].

The limitations of our study include those which are inherent to observational studies. Although the size of our study population was rather small, it was representative of real-life T2DM patients from Poland with relatively good glycaemic control. Moreover, the study was sufficiently powered to show the difference in cfDNA between patients with and without MI based on a previous study in T2DM patients [17]. Since we assessed glycaemia once, we did not assess potential links of NETosis with varying plasma glucose in T2DM, especially with episodes of hypoglycaemia, which is known to contribute to a worse prognosis, resistance to lysis, and compact fibrin clot formation [41]. All reported associations cannot be necessarily considered as cause-and-effect relationships, however, they are largely in line with in vitro data published in the literature [14, 15]. We did not assess the extent of oxidation as a potential modifier of NETosis and fibrin properties in this study [42]. A large prospective study with a long-term follow-up would be required to assess whether NETosis and its association with the prothrombotic blood alterations reported here, is related to adverse clinical events, including cardiovascular death, in T2DM.

## Conclusions

In T2DM, NETosis markers in circulating plasma, such as H3Cit and cfDNA, are related to glycemia control, systemic low-grade inflammation markers and previous MI. Enhanced NETosis detectable in circulating blood is associated with a prothrombotic state, especially hypofibrinolysis in T2DM patients. The present study shows that NETosis might contribute to thrombotic and cardiovascular risk in that disease. A role of NETs generation in the natural course of diabetes and its complications deserves further investigations.

## Abbreviations

aPTT: activated partial thromboplastin time
AUC: area under the curve
CAD: coronary artery disease
CAT: calibrated automated thrombogram
cfDNA: cell-free deoxyribonucleic acid
CVD: cardiovascular disease
HbA1c: glycated hemoglobin
GFR: glomerular filtration rate
H3Cit: citrullinated histone H3
hs-CRP: high-sensitivity C-reactive protein
IL-6: interleukin 6
IL-8: interleukin 8
MI: myocardial infarction
NE: neutrophil elastase
NETs: neutrophil extracellular traps
OR: odds ratio
PAD: peripheral artery disease
PAI-1: plasminogen activator inhibitor-1
TAFI: thrombin activatable fibrinolysis inhibitor
TG: thrombin generation
T2DM: type 2 diabetes mellitus
CI: confidence interval
